# Round the Hospitals

**Published:** 1916-05-06

**Authors:** 


### ROUND THE HOSPITALS.

The children's ward where the management is of the best is a valuable asset for a voluntary hospital. We have received the following from a matron of a children's hospital, which may, we think, prove of interest and value to other matrons elsewhere: For many years one of my most pleasant duties has been the providing of clothing for my large family of hospital babies. In this department my committee have always given me a perfectly free hand in regard to materials and cost. In my opinion not only for underclothing and nighties, but for young children's frocks, nothing can equal viyella. It is soft and warm, and yet will not irritate the most tender skin. It washes well, does not shrink, and (if the more delicate shades be avoided) the colours will withstand the treatment of the average laundry. Unlike flannels, nun's-veiling, etc., cream viyella does not turn a dirty-looking yellow. Lastly, it wears well. The medium weight known as TT I prefer for all-round purposes. In colours it is made only in 31 inches material, price 1s. 11½d. per yard. In cream it may be had 36 inches wide at 2s. 6d., 44 inches at 2s. 11d., and 54 inches at 3s. 9d. For underclothing I like white, cream, or very pale natural. For the baby frocks cream or pink is most suitable. Pale blue is pretty but fades. For bigger children, crimson, navy, olive-green, and golden-brown I found to be serviceable colours. But there is a very large selection of shades to choose from, and each matron can suit her own individual taste. Viyella bootees I find preferable to any other make.

---

Our correspondent proceeds: When I first adopted viyella for my children people were horrified at my extravagance. They agreed that it was very beautiful, but in an institution something cheaper might have served the purpose. Some even whispered that it was much too beautiful for poor children. Why, I ask, should institution children have to submit to the indignity of navy drill and turkey twill? The first is stiff and uncomfortable when new, and the dye rubs off and finally washes out. The latter always has a cold clammy feeling. All children (especially girls) love pretty clothes, and I believe in developing a child's æsthetic tastes even in the matter of apparel. The ages of my children who wear viyella range from two weeks to five years. The bigger ones play outside the greater part of the day, and their frocks are subjected to rough wear and are constantly in the wash. After the first year's wear the elbows required patching. At the end of another year I had the frocks re-sleeved. After a third year, when sleeves were again in need of repair and the skirts also thin, I issued new frocks. The thicker parts of the old ones were kept for patches, the thin parts for polishing rags. During the hotter weather, for about four months the children wear cotton frocks. Thus the viyella dresses, after three years in use, had been subjected to two years' continuous wear. Can anyone call that extravagant? We invite correspondence, and shall welcome news of the children's summer frocks, their pinafores, and their foot-gear.

---

The theoretical training received by the nurses at the Westminster Hospital may be taken as typical of what may be called the first-line hospitals. There is a course of twelve lectures given by the matron from October to Christmas, followed by an examination. A course of lectures on surgical nursing, given by the senior surgeon, from January to March. The nurses are coached for the surgical examination in small classes of eight to ten by sisters who are present at the lectures, and are prepared to answer questions and elucidate difficulties. Invalid cookery demonstrations, instrument classes, and practical lessons on bandaging occupy the second year, and in March a course of medical lectures for second-year nurses is followed by a final examination, which includes a practical *viva voce*, for which prizes are given. The lectures and classes, except those for cookery, are given between 8 and 9 in the evening; those for invalid cookery take place in the morning. All examinations are conducted by outside examiners, who are not personally known to the nurses. For the practical nursing and surgical examination the examiner is Mr. Russell Howard, hon. surgeon to the London Hospital.

---

The whole nation, and especially workers everywhere, are settling down to war conditions and nobly doing their utmost to help. This is especially true of the voluntary hospitals. Salisbury Infirmary, for instance, which had 108 beds before the war, now has 200 beds in all. All the official work is done at the infirmary by voluntary helpers, a room being set apart and fitted as a Red Cross office. Referring to the foreword in this department last week, the matron states as her experience that the one thing that helps all hospital workers in these days of war is the presence of wounded warriors. Practically every member of the staff has relations in the Services, so all labour under the strain of anxiety, and some of bitter sorrow. It is good, too, for the civilian patients to come in contact with the wounded, to witness their courage and the way they make light of their troubles. Another result has been that interest in the hospital and in many branches of its work has greatly increased, so we hope the treasurer may receive all the money necessary to meet the increased cost of maintenance. To us Salisbury Infirmary is always an interesting place to visit, for it is an old county hospital where the administration is kept alive and active. Then, too, the matron's own room and what it contains have an inspiring effect upon workers who know and feel.

---

The scheme for an Imperial Nurses' Club is hanging fire. Some regret this, for they hold there is a need for such an institution. Not only nurses from overseas but those trained or training in London would benefit, it is held, from the advantage of a meeting-place in a convenient locality, where large, airy, well-furnished rooms, good catering at moderate prices, and other comforts offered by ladies' clubs should be available. It is believed that many nurses would pay a guinea a year to belong to such a club, which would serve to break down barriers between different sections of the profession and promote good feeling. It would, it is contended, prevent much of the loneliness and isolation which is now an unpleasant feature of the life of a nurse in London. So whatever the fate of the present proposals, an Imperial Club for Nurses in London will one day, no doubt, find its place amongst social institutions.

---

*** It is our invariable practice to pay for all items of news which we may publish. The minimum payment is 5s. Paragraphs of about twenty lines in length—that is to say, of 150 words or less—are preferred. In this section during the war we propose to give 10s. to the correspondent who may send us in any week the best incident connected with hospital life and work. In this way we hope to afford to all practical aid and encouragement; while those who reciprocate will be able to render us invaluable service. Contributions should be addressed to The Editor, The Hospital, 28 and 29 Southampton Street, Strand, London, W.C., and, to be in time for the current week's issue, should reach him by the first post on Mondays.

For Appointments see page 138; The Laundry, see page 126.

